# Genetic Variation in the Epidermal Transglutaminase Genes Is Not Associated with Atopic Dermatitis

**DOI:** 10.1371/journal.pone.0049694

**Published:** 2012-11-26

**Authors:** Agne Liedén, Mårten C. G. Winge, Annika Sääf, Ingrid Kockum, Elisabeth Ekelund, Elke Rodriguez, Regina Fölster-Holst, Andre Franke, Thomas Illig, Maria Tengvall-Linder, Hansjörg Baurecht, Stephan Weidinger, Carl-Fredrik Wahlgren, Magnus Nordenskjöld, Maria Bradley

**Affiliations:** 1 Department of Molecular Medicine and Surgery, Karolinska Institutet, Stockholm, Sweden; 2 Dermatology Unit, Department of Medicine Solna, Karolinska University Hospital, Stockholm, Sweden; 3 Department of Clinical Neurosciences, Karolinska Institutet, Stockholm, Sweden; 4 Department of Dermatology, University of Kiel, Kiel, Germany; 5 Institute of Clinical Molecular Biology, Christian- Albrechts-Universität zu Kiel, Kiel, Germany; 6 Research Unit of Molecular Epidemiology, Helmholtz Zentrum München, German Research Center for Environmental Health, Neuherberg, Germany; 7 Hannover Unified Biobank, Hannover Medical School, Hannover, Germany; 8 Clinical Immunology and Allergy, Department of Medicine Solna, Karolinska Institutet, Stockholm, Sweden; University Hospital Hamburg-Eppendorf, Germany

## Abstract

**Background:**

Atopic dermatitis (AD) is a common chronic inflammatory skin disorder where epidermal barrier dysfunction is a major factor in the pathogenesis. The identification of AD susceptibility genes related to barrier dysfunction is therefore of importance. The epidermal transglutaminases (*TGM1, TGM3* and *TGM5)* encodes essential cross-linking enzymes in the epidermis.

**Objective:**

To determine whether genetic variability in the epidermal transglutaminases contributes to AD susceptibility.

**Methods:**

Forty-seven single nucleotide polymorphisms (SNPs) in the *TGM1, TGM3* and *TGM5* gene region were tested for genetic association with AD, independently and in relation to *FLG* genotype, using a pedigree disequilibrium test (PDT) in a Swedish material consisting of 1753 individuals from 539 families. In addition, a German case-control material, consisting of 533 AD cases and 1996 controls, was used for *in silico* analysis of the epidermal *TGM* regions. Gene expression of the *TGM1, TGM3* and *TGM5* gene was investigated by relative quantification with Real Time PCR (qRT-PCR). Immunohistochemical (IHC) analysis was performed to detect TG1, TG3 and TG5 protein expression in the skin of patients and healthy controls.

**Results:**

PDT analysis identified a significant association between the *TGM1* SNP rs941505 and AD with allergen-specific IgE in the Swedish AD family material. However, the association was not replicated in the German case-control material. No significant association was detected for analyzed SNPs in relation to *FLG* genotype. TG1, TG3 and TG5 protein expression was detected in AD skin and a significantly increased *TGM3* mRNA expression was observed in lesional skin by qRT-PCR.

**Conclusion:**

Although *TGM1* and *TGM3* may be differentially expressed in AD skin, the results from the genetic analysis suggest that genetic variation in the epidermal transglutaminases is not an important factor in AD susceptibility.

## Introduction

Atopic dermatitis (AD, OMIM#603165) also referred to as eczema [1], is a common chronic inflammatory skin disorder which results from a complex interaction of genetic and environmental factors [2], [3], [4]. Epidermal barrier dysfunction is a major component in the development of AD [2], most recently highlighted by the identification of the filaggrin (*FLG*) gene as a susceptibility gene in AD [5]. Filaggrin aggregates keratin intermediate filaments in the cornified envelope and is also believed to play additional roles in the formation of a functional epidermal barrier. However, a number of genes are likely to be responsible for the barrier dysfunction seen in AD patients and the identification of these genes would improve the understanding of AD pathogenesis and provide an important basis for improved therapeutics in AD.

Transglutaminases (TGs) are Ca^2+^-dependent enzymes that catalyze the formation of Nε-(γ-glutamyl) lysine bonds between proteins and the covalent incorporation of biogenic polyamines into proteins through N,N-bis(γ-glutamyl) bonds. The TGs are important in many biological processes including the formation of the epidermal skin barrier [6]. Among the nine mammalian TGs, TG1, TG3 and TG5 are expressed in the epidermis and are known to be involved in the formation of the cornified cell envelope [7]. TGs are responsible for the cross linking of several structural proteins including envoplakin, periplakin, loricrin, small proline-rich proteins and the previously mentioned filaggrin protein. TG1 are also capable of attaching and cross link lipids on the already cross linked proteins [7].

Rare mutations in TGs have been identified in severe recessive epidermal disorders, with mutations in the *TGM1* gene causing lamellar ichthyosis [8], [9] and mutations in the *TGM5* gene causing the acral form of “the peeling skin syndrome” [10]. Furthermore, in a previously published cDNA microarray study we showed increased expression of the *TGM1* and *TGM3* transcripts in the skin of AD patients sensitized to skin-colonizing yeast *Malassezia sympodialis (Mal s)* [11].

Although TGs are key players in forming the cornified envelope, and are linked to epidermal disorders, and map in genomic regions (14q12, 20p13 and 15q15) previously linked to AD and associated phenotypes [12], [13], [14], a more detailed study investigating a potential role in AD pathogenesis has to our knowledge not been performed. We therefore decided to test whether genetic variation at the *TGM1, TGM3* and *TGM5* gene loci might be associated with AD susceptibility and to study the expression of these genes in the skin of AD patients and healthy controls.

**Figure 1:**
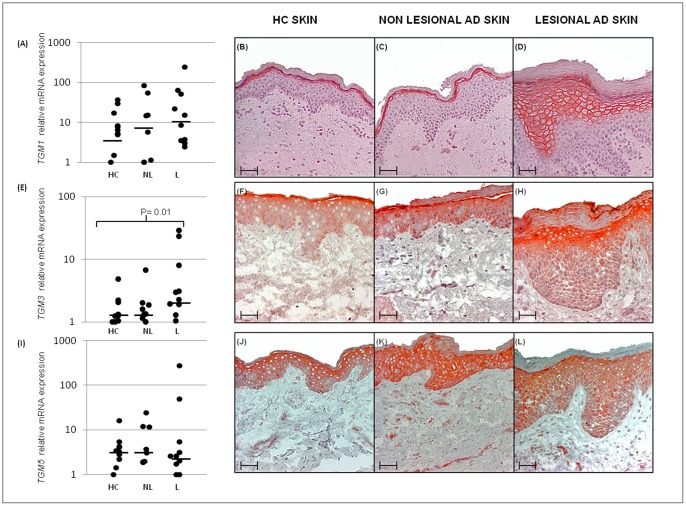
*TGM1*, *TGM3* and *TGM5* gene expression in the skin of AD patients and healthy controls. TGM transcript levels (A, E and I) of healthy controls (HC, n = 10) non lesional skin of AD patients (NL, n = 7) and lesional skin from AD patients (L, n = 10). Horizontal bars represent median values in each group and data is presented on a logaritmic scale. For IHC analysis of the TG protein expression, skin sections from nine AD patients and ten healthy controls were stained. Representative staining from one healthy control and one patient is shown in the figure for TG1 (B–D), TG3 (F–H) and TG5 (J–L) expression. Scale bar represents 50 µm.

## Materials and Methods

### Genetic association analysis in the Swedish family material

The material consisted of 1753 individuals from 539 nuclear families with at least two AD affected sibs in each family and has been described previously [15], [16]. Families including a sibling with allergen-specific IgE was used to form a subgroup (AD^IgE+^) in the analysis (n = 404). All patients in this subgroup had raised specific IgE against single or a panel of common aero-allergens (reported as positive or negative), using Phadiatop analysis (Phadia, Uppsala, Sweden).

Genotype data for SNPs in the *TGM1, TGM3* and *TGM5* gene region was downloaded from the HapMap project (release #23a, NCBI build 36, dbSNP b126). Selection of SNPs was mainly done by using the Tagger feature in Haploview program [17] with a minor allele frequency of 5% as cut-off. The pair wise and 2- and 3-marker tagging option was used with an r^2^ threshold of 0.8. Genotyping was performed in two sets. The first set of SNPs was selected to cover the *TGM1* locus and typed with TaqMan® SNP Genotyping Assays (Applied Biosystems, Foster City, CA, USA). The second set included additional SNPs for the *TGM1* locus, included due to the LD pattern in this region, and SNPs covering the *TGM3* and *TGM5* loci. The second set was typed on a MALDI-TOF (Matrix Assisted Laser Desorption-Ionisation-Time Of Flight) platform (Sequenom). Information regarding the Sequenom methodology is provided elsewhere [18]. Population Hardy-Weinberg equilibrium was evaluated using the Haploview program and families with Mendelian errors were excluded. A complete list of SNPs, including quality assessment, is available in Table S1. In addition, association of all analyzed SNPs was compared to *FLG* genotype. Siblings in the family material were sub-grouped into either *FLG* wildtype (n = 998) or *FLG* heterozygote/compound heterozygote/homozygote (n = 277), determined by previously published genotype data for the study population for mutations R501X and 2282del4 [19] combined with genotype data for R2447X and S3247X determined with previously described primers and PCR conditions [20].

### In silico genetic association analysis in the German case-control material

SNP data from the *TGM1* region were extracted from a recently published genome-wide association study (GWAS) [21]. Variants within 100 kb up- or downstream of the candidate region were extracted and LD was investigated in the CEU population HapMap release 28 [22]. *In silico* analysis was performed for 533 AD cases (of which 335 had AD ^IgE+^), recruited in Munich and Kiel, Germany, and 1996 healthy controls from the population-based KORA S4/F4 survey [23]. SNPs were filtered according to call rate>0.97, HWE deviation p>0.001 and minor allele frequency (MAF) in controls >0.05.

### Gene expression and immunohistochemical analysis

For relative quantification with Real Time PCR (qRT-PCR), skin biopsies from 10 adult patients with AD and 10 healthy controls were collected and for the immunohistochemical analysis (IHC), skin biopsies from 10 adult patients with AD and 9 healthy controls were collected at the Department of Dermatology, Karolinska University Hospital Solna, Stockholm, Sweden. Punch biopsies from non-lesional AD skin and from healthy control skin were taken from the lower back region, whereas biopsies from lesional skin were taken from available areas with comparable skin thickness. Inclusion criteria for the AD patients were diagnosis according to the UK working party criteria [24]. The patients had not received treatment for the previous two months. All patients had raised specific IgE against a panel of common aero-allergens, Phadiatop analysis (Phadia, Uppsala, Sweden). The healthy controls had no clinical symptoms or history of allergy or skin diseases, had total serum IgE levels <122 kU/l, and were Phadiatop negative (<0.35 kU/l).

Biopsies were snap frozen and stored at −80°C. For IHC, six μm cryo sections were fixed in acetone, and blocked with 0.3% H_2_O_2_, normal goat serum (dilution 1/10) and avidin and biotin (Vector Laboratories Inc. Burlingame, CA, USA). A mouse monoclonal antibody against human TG1, dilution 1/250 (Biogenesis, Einköpngland, UK), was used for staining and a biotinylated horse-anti-mouse secondary antibody (dilution 1/400, Vector Laboratories Inc.) was used as a secondary antibody. TG3 and TG5, were detected by a goat polyclonal antibody against human TG3, dilution 1/200 (Santa Cruz, CA, USA), and a rabbit polyclonal antibody against human TG5, dilution 1/4000 (Novus, Cambridge, UK), respectively, and biotinylated horse-anti-goat and biotinylated goat-anti-rabbit (dilution 1/200, Vector Laboratories Inc.) were used as secondary antibodies. The sections were then incubated with preformed avidin-biotin-enzyme complex (ABC-ELITE reagent, Vector laboratories Inc.) and developed with 3-amino-9-ethylcarbazole (AEC) substrate. Counterstaining was made with Mayer's haematoxylin. Irrelevant mouse IgG2a was used as a control antibody. The results from the IHC analysis, including differences in staining between samples, were evaluated independently by two dermatologists (MB and CFW).

Total RNA was extracted from skin biopsies with the Trizol Reagent (Invitrogen, Carlsbad, CA, USA) and used for gene expression analysis. Total RNA quality was assessed by NanoDrop spectrophotometer and gel electrophoresis. One microgram of total RNA was used for cDNA synthesis with the SuperScript III System (Invitrogen) using random hexamers and oligo (dT) primers. qRT-PCR was performed using 18S as endogenous control, Power SYBR Green and the 7900HT Fast Real-Time PCR System (Applied Biosystems). Primer sequences are listed in table S2.

### Statistical analysis

PDT and odds ratio (OR) estimates for the genetic association study conducted in the family material was performed using the Unphased (3.1.3) program [25]. Statistical power analysis was performed using the Genetic Power Calculator [26]. The statistical power was estimated to be above 80% for detecting a factor with an allele frequency of 0.10 and an OR of 1.5 in the family material. The case-control analysis was carried out with PLINK [27] using a chi-square test for the two by two table for each SNP [21]. For qRT-PCR analysis the Mann-Whitney U test was performed to evaluate difference in expression between the sample groups. P-values <0.05 were considered significant.

### Ethics

All studies were approved by the local ethics committee, conducted according to the Declaration of Helsinki principles, and the subjects gave their written informed consent.

## Results

### Genetic association of the TGM1 gene in the Swedish family material

Genotyping was performed for 47 SNPs in total covering the *TGM1, TGM3* and *TGM5* locus (a complete list of SNPs is supplied in table S1) in a Swedish family material consisting 1753 individuals from 539 nuclear families. The success rate was above 85.5% for all genotype assays. The first set of eight SNPs selected for genotyping, with TaqMan® SNP Genotyping Assays, targeted the *TGM1* region. Analysis of the first set of *TGM1* SNPs identified one SNP in the 5′ region, rs941505, that was significantly associated with AD ^IgE+^, p = 0.002, showing an estimated OR of 0.60 (confidence interval (CI), 0.43–0.84) for the minor allele. Data from the HapMap CEU population indicates some level of LD between rs941505 and upstream SNPs. Additional SNPs in this region were therefore genotyped, with Sequenom methodology, in the second set of SNPs that also targeted the *TGM3* and *TGM5* region. Genotyping for the associated SNP rs941505 was also repeated with the Sequenom methodology, validating the results of TaqMan run. Analysis in RAVEN (http://www.cisreg.ca/cgi-bin/RAVEN/a) and the MAPPER database suggest that the rs941505 SNP could alter putative binding site of transcription factors overlapping this position. None of the SNPs analyzed in the *TGM3* and *TGM5* region provided p-values that would remain significant after correction for multiple testing (a complete list of p-values is presented in table S1). Similarly, no significant association remained after multiple testing of analyzed SNPs in relation to *FLG* genotype (data not shown).

### In silico genetic association analysis in the German case-control material

According to data from the HapMap CEU population the *TGM1* SNP rs941505 SNP is in complete LD (r^2^ = 1) with one SNP, rs2075530, previously analyzed in a recently published GWAS [21]. Also, the minor allele frequency for the rs2075530 SNP in the German material was the same as for the rs941505 SNP in the Swedish material (∼0.095), i.e. in support of complete LD between these SNPs. However, *in silico* association analysis did not show any significant association with this SNP with AD (p = 0.96) or a subgroup of AD ^IgE+^ patients (p = 0.80).

### Increased TGM3 mRNA levels in lesional AD skin

Expression of the *TGM1, TGM3 and TGM5* gene was measured by qRT-PCR and the results showed a significantly higher level of *TGM3* mRNA in lesional skin from AD patients (n = 10) compared to skin from healthy controls, p = 0.01. Further, a median increase of *TGM1* expression was noted in both non-lesional and lesional skin (Fig. 1). Four out of the seven samples, where a paired biopsy was available, showed a ∼2-fold increase (or more) for *TGM1* when comparing lesional to non-lesional samples. However, this trend did not reach statistical significance. Due to alternate splicing, expression of the *TGM5* gene was evaluated with two primer pairs specific for the different transcript isoforms. The results were very similar for both isoforms (data not shown) with no significant difference between the sample groups. The result for isoform 1 is presented in Figure 1.

### TG1, TG3 and TG5 protein expression in AD skin

Finally, IHC analysis was used to study the expression of the TG1, TG3 and TG5 protein in lesional and non-lesional skin of AD patients and in healthy individuals. The results indicated a distinct TG1 and TG3 expression in a majority of lesional skin samples, while non-lesional skin samples appeared to have a less marked expression of these proteins compared to healthy controls (Fig. 1). Furthermore, in lesional skin, characterized by hyperplasia, TG1 and TG3 expression was found in several of the suprabasal layers, while in skin from healthy individuals the proteins was localized in the outermost granular layer of the epidermis. No apparent differences regarding TG5 expression was found.

## Discussion

Epidermal barrier dysfunction is an important factor in AD pathogenesis and the identification of susceptibility genes in barrier dysfunction is therefore of major importance. The epidermal transglutaminases, *TGM1, TGM3* and *TGM5* encodes essential cross-linking enzymes in the epidermis and map in genomic regions that have previously been linked to AD and are therefore strong candidate genes for AD.

In this study we tested whether genetic variability at the epidermal transglutaminase loci may contribute to AD susceptibility and investigated gene expression in AD patients and healthy controls. We detected a significant genetic association for one SNP, rs941505, located upstream of the *TGM1* gene in the 14q12 region in a putative transcription binding site. The minor allele was under-transmitted to offspring with AD and allergen-specific IgE (OR = 0.60). To replicate our finding in an independent material, we used a German case-control material, previously used in a GWAS exploring AD in the European population [21]. Looking at the SNPs present on the arrays used in the GWAS, we conclude that the SNP rs2075530 in high LD with rs941505 were not significantly associated with AD or the subgroup with allergen-specific IgE.

Our observations from expression data indicate a marked protein expression of both TG1 and TG3 in the skin of AD patients compared to the skin of healthy controls. Also, although the increase of *TGM1* transcript levels unlike *TGM3* were non-significant, median expression was higher in both non-lesional and lesional skin compared to healthy skin. The limited sample size might explain why the levels did not reach statistical significance. Both TG1 and TG3 are thought to cooperatively cross-link proteins involved in CE formation residues at different cellular sites combining intramolecular crosslinks (*TGM3*) and formation of larger oligomers (*TGM1*) during cornification [28], [29]. *TGM1* and *TGM3* are both expressed in the granular and spinous layers of the epidermis, but with more limited *TGM3* expression [30]. Interestingly, a recent study show reduced expression of *TGM3* in AD skin compared to control skin [31]. However, this study investigates protein extracted from stratum corneum, whereas our data represent semi-quantification based on all epidermal immunolocalization. It is possible that differences in expression and function vary based on epidermal localization, which cannot be excluded in our data.

An elevated *TGM1* expression would be in line with results from a previous study where we showed a significant increase in the expression of the *TGM1* mRNA and protein in the skin of AD patients sensitized to *Mal s* compared to healthy controls [11]. Furthermore, a recently published study has shown that *TGM1* and *TGM3* were up regulated in AD skin upon barrier disruption using a tape-stripping technique [32]. A disrupted barrier is evident in AD skin and may be concordant with our findings. The distinct expression observed in lesional AD skin may, at least in part, indicate that *TGM1* and *TGM3* activity may be susceptible to inflammatory mediators. This hypothesis is supported by previous data, showing that *TGM1* is susceptible to up regulation following pro-inflammatory cytokine stimulation [33]. However, it may be more likely that the observed expression patterns reflect epidermal hyper proliferation and/or a impaired differentiation process, and would be in line with the increase in TG1 that has been noted in other hyper-proliferatory disorders such as psoriasis [34].

In conclusion, although *TGM1* and *TGM3* may be differentially expressed in AD skin, the results from the genetic analysis suggest that genetic variation in the epidermal transglutaminases is not an important factor in AD susceptibility.

## Supporting Information

Table S1 **Genotyped SNPs.** Positions are from dbSNP build 126, UCSC NCBI36/hg18. SNPs that failed quality assessment and were replaced have been omitted from the table. Please note that two SNPs, rs7151201 and rs941505, were re-typed on the Sequenom platform. Two replacement SNPs were from dbSNP with no HapMap data available (marked with N/A in the column for HapMap concordance). HWpval  =  Hardy-Weinberg equilibrium p-value calculated using Haploview. HapMap concordance rates were calculated by typing 40 individuals with known genotypes from the HapMap project. Furthermore, concordance rates were also evaluated by re-typing a set of 90 in house control samples (Mutation analysis facility, Karolinska Institutet). Presented are uncorrected p-values for all typed SNPs, AD  =  Atopic dermatitis, AD^IgE+^  =  Atopic Dermatitis with allergen-specific IgE (positive in Phadiatope testing).
(DOCX)

Table S2 **Primers for Real-Time PCR.**
(DOCX)
