# Supplementary material for: Genetic Variation in the Epidermal Transglutaminase Genes Is Not Associated with Atopic Dermatitis
Source: PLoS One. 2012 Nov 26;7(11):e49694. doi: 10.1371/journal.pone.0049694 (PMC3506648; doi:10.1371/journal.pone.0049694)
Supplement: Table S1 — Genotyped SNPs. Positions are from dbSNP build 126, UCSC NCBI36/hg18. SNPs that failed quality assessment and were replaced have been omitted from the table. Please note that two SNPs, rs7151201 and rs941505, were re-typed on the Sequenom platform. Two replacement SNPs were from dbSNP with no HapMap data available (marked with N/A in the column for HapMap concordance). HWpval = Hardy-Weinberg equilibrium p-value calculated using Haploview. HapMap concordance rates were calculated by typing 40 individuals with known genotypes from the HapMap project. Furthermore, concordance rates were also evaluated by re-typing a set of 90 in house control samples (Mutation analysis facility, Karolinska Institutet). Presented are uncorrected p-values for all typed SNPs, AD = Atopic dermatitis, ADIgE+ = Atopic Dermatitis with allergen-specific IgE (positive in Phadiatope testing). (DOCX) [file pone.0049694.s001.docx]

**Supplementary table S1: Genotyped SNPs**
