# Supplementary material for: Genetic Variation in the Epidermal Transglutaminase Genes Is Not Associated with Atopic Dermatitis
Source: PLoS One. 2012 Nov 26;7(11):e49694. doi: 10.1371/journal.pone.0049694 (PMC3506648; doi:10.1371/journal.pone.0049694)
Supplement: Table S2 — Primers for Real-Time PCR. (DOCX) [file pone.0049694.s002.docx]

**Supplementary table S2: Primers for Real-Time PCR**

| **Primer** | **Sequence (5’-3’)** |
| --- | --- |
| 18S_F | CGGCTACCACATCCAAGGAA |
| 18S_R | GCTGGAATTACCGCGGCT |
| TGM1_F | CCCCAGTGCAAGTGAAGACT |
| TGM1_R | CCAAGATAGGGGCACACTACA |
| TGM3_F | TTGGGTAACCTGAAGATCGAC |
| TGM3_R | GAGAAGTCGGCGAGCAGTT |
| TGM5_isoform1_F | CTGCTTTTCAATCCCTGGTG |
| TGM5_ isoform1_R | ATCCAGTTCTTGCTGCCTTG |
| TGM5_isoform2_F | TTCGTGGTTGAAACTGAGGA |
| TGM5_ isoform2_R | GCTGCCTTGGTAGATGAAGC |
